# Estimating Human Fat and Muscle Conductivity From 100 Hz to 1 MHz Using Measurements and Modelling

**DOI:** 10.1002/bem.22541

**Published:** 2025-01-18

**Authors:** Otto Kangasmaa, Ilkka Laakso, Gernot Schmid

**Affiliations:** ^1^ Department of Electrical Engineering and Automation Aalto University Espoo Finland; ^2^ Seibersdorf Labor GmbH Seibersdorf Austria

**Keywords:** anisotropic muscle, bioimpedance, low frequency, tissue electrical properties

## Abstract

The electrical conductivity of human tissues is a major source of uncertainty when modelling the interactions between electromagnetic fields and the human body. The aim of this study is to estimate human tissue conductivities in vivo over the low‐frequency range, from 30 Hz to 1 MHz. Noninvasive impedance measurements, medical imaging, and 3D surface scanning were performed on the forearms of ten volunteer test subjects. This data set was used to create subject‐specific forearm models, numerically solve an electrostatic forward problem, after which the tissue conductivities could be estimated by solving a probabilistic inverse problem. The electrical conductivity of skeletal muscle was found to be highly anisotropic at frequencies below 10 kHz, with conductivities of 0.13 (95% credible interval (CrI): 0.10–0.16) S/m perpendicular and 0.56 (CrI: 0.52–0.60) S/m parallel to the muscle fibre direction. This anisotropy decreased with increasing frequency with these values being 0.65 (CrI: 0.48–1.00) S/m and 0.78 (CrI: 0.72–0.85) S/m at 1 MHz. The conductivity of subcutaneous fat was found to be almost constant across the considered frequency range, with values of 0.21 (CrI: 0.12–0.31) S/m and 0.22 (CrI: 0.07–0.37) S/m at 10 kHz and 1 MHz, respectively. Our study provides robust uncertainty bounds for human tissue conductivity values, which are crucial in the computational assessment of human electromagnetic field exposure. Additionally, our findings are applicable to other fields of modelling such as medical stimulation or measurement technologies.

## Summary

- Noninvasive impedance measurements, anatomical forearm models, and a probabilistic estimation method were used to determine the electrical conductivity of skeletal muscle and fat in vivo.
- Skeletal muscle was found to be electrically anisotropic, with the anisotropy decreasing with increasing frequency, whereas the conductivity of fat stayed constant over the considered frequency range.
- The estimated conductivities have robust uncertainty bounds which were further used to study how uncertainty in conductivities propagates into induced electric fields in a magnetic field exposure scenario.

## 1 Introduction

Low‐frequency time‐varying electromagnetic fields (⪅ 1 MHz) may stimulate sensory neurons or activate muscle cells. To avoid these adverse health effects, guidelines (ICNIRP 2010) and standards (IEEE‐C95.1 2019) have been created to give guidance on the safe use of electromagnetic energy. These guidelines and standards define frequency‐dependent restricting quantities that limit the strength of the induced electric field inside the human body. In some situations, a more thorough exposure assessment is needed. Since the internal electric field strength cannot be measured directly inside the human body, computational dosimetry offers a way to assess the exposure. These dosimetric calculations require the use of computer models that represent the human anatomy. A major source of uncertainty associated with these models is the electrical conductivity values assigned to the model tissues. Furthermore, modelling electrically anisotropic tissues as isotropic increases this uncertainty.

Skeletal muscle and fat are the two most abundant tissue types by weight (ICRP 2002). Therefore, the dielectric properties of these tissues have gained the attention of the scientific community at regular intervals and have been studied extensively. However, at low frequencies, the reported values show large variability between studies. For example at 1 kHz, the conductivity of fat ranges from 0.02 S/m (Gabriel, Lau, and Gabriel 1996) to 0.41 S/m (Stoneman et al. 2007), and the conductivity of skeletal muscle ranges from 0.05 S/m (Hart, Berner, and McMillen 1999) to 0.19 S/m (Gabriel, Peyman, and Grant 2009) and 0.09 S/m (Hart, Berner, and McMillen 1999) to 0.56 S/m (Epstein and Foster 1983) lateral‐ and longitudinal to the muscle fibres, respectively. The reason for these large discrepancies may result from, for example, differences in measurement methods, heterogeneity and potential anisotropy of targeted tissues, sample species, and temperature change. Moreover, the majority of studies, researching the dielectric properties of tissues, have performed their measurements on excised tissue samples, which has been shown to drastically alter the measured values (Ørjan G Martinsen, Grimnes, and Mirtaheri 2000; Schmid et al. 2003). The conductive properties are also dependent on frequency, further adding to the complexity of the problem. Ideally, tissue conductivities would be measured from live humans without any invasive procedures over a wide frequency range.

To tackle this challenge, electrical impedance tomography (EIT) offers a methodology to image conductivity distributions non‐invasively. EIT uses surface impedance measurements to determine a map of the volume conductivity. Unfortunately, solving the conductivity distribution from these surface impedance measurements is an ill‐posed and ill‐conditioned inverse problem, which results in poor spatial resolution. This makes EIT a somewhat unsuitable method to derive tissue conductivities. However, adding knowledge of tissue boundaries simplifies the problem. Bounded EIT (bEIT) has been mainly used to estimate scalp and skull conductivities and study the skull‐to‐brain conductivity ratio (Oostendorp, Delbeke, and Stegeman 2000; Goncalves et al. 2003; Clerc et al. 2005; Dabek et al. 2015; Fernández‐Corazza et al. 2018). In our previous work (Kangasmaa and Laakso 2022), we estimated the conductivity of anisotropic skeletal muscle and fat from human legs. Our method deviated from the aforementioned bEIT studies, such that conductivities were solved for a population rather than for individual subjects/models. However, that study had some limitations, namely a limited frequency range (10–100 kHz) and a simplified representation of muscle anisotropy.

Therefore, this study was designed to provide new information on the low‐frequency conductivities of human tissues that extend down to power line frequencies. The conductivity of anisotropic muscle and subcutaneous fat was estimated using a method similar to bEIT. First, we performed noninvasive impedance measurements, magnetic resonance imaging (MRI), and 3D surface scanning on the forearms of 10 test subjects. Second, subject‐specific anatomical forearm models were created based on this gathered data set. Third, the electrostatic forward problem was solved numerically using the finite element method (FEM). Finally, the conductivity and its uncertainty were estimated by solving a probabilistic inverse problem. These estimates were also used to study how their uncertainty propagates into induced electric fields when a forearm model is exposed to a localized magnetic field.

## 2 Materials and Methods

### 2.1 Study Participants

Ten healthy subjects (six male and four female) volunteered to participate in this study. Their ages ranged from 22 to 42, with a mean age of 32 and a standard deviation of 6. The experimental procedure was explained to each subject, after which they gave their written consent for participation. The study was approved by the Aalto University Research Ethics Committee (diary number D/1545/03.04/2023).

### 2.2 Impedance Measurements

Impedance measurements were performed on the right forearm of each test subject. Eight standard electrocardiography electrodes (3M Red Dot 2238 Soft Cloth Monitoring Electrodes, 3M, Maple‐wood, Minnesota, USA) were used as the interface between the impedance analyser (MFIA Impedance Analyzer, Zurich Instruments AG, Zurich, Switzerland) and the test subject. A tetrapolar (four electrode) configuration was used for the measurements, where the current was fed from two electrodes and voltage was measured from the remaining two. As the stimulus, the impedance analyser was set to apply a 1 V excitation voltage from 30 Hz to 1 MHz, at 46 logarithmically spaced points. This frequency sweep was performed bidirectionally, measuring at each frequency twice to reduce the uncertainty of the measurement.

**Figure 1:**
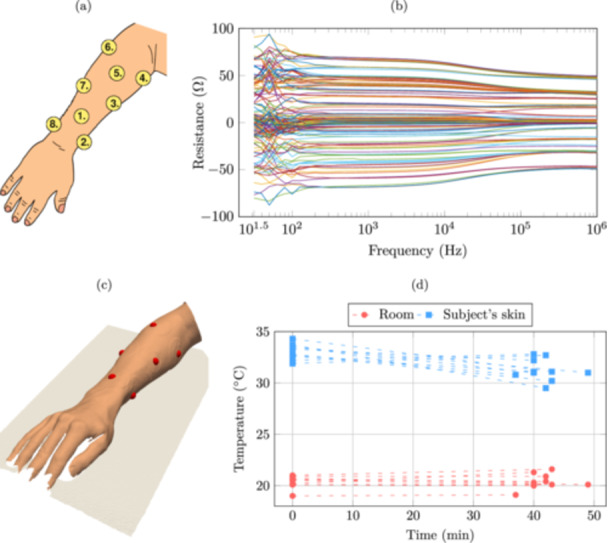
(a) Approximate measurement electrode locations on the forearm. Data from an example test subject: (b) real part of the impedance from all measurements and (c) 3D surface scan with electrode locations marked in red. (d) Room and skin temperature measurements before and after the impedance measurements.

During the measurements, the subjects were asked to keep their right arm on a table, palm facing down and relaxed. The electrodes were placed between the ulnar styloid process (the ‘bony knob’ on the wrist) and the elbow so that each electrode was still accessible for measurement without the subject moving their arm. An illustration of the approximate electrode locations is shown in Figure 1a. With eight measurement electrodes, there exist 82=28 unique current feeding configurations. Choosing one of the remaining electrodes as a voltage reference, five voltage measurements can be performed for each current feeding configuration. This resulted in 140 impedance measurements performed on each subject. Figure 1b shows the real part of the impedance from all the measurements of an example test subject.

The locations of the measurement electrodes were recorded with a handheld 3D surface scanner (Artec Leo, Artec 3D, Senningerberg, Luxembourg). An example surface scan is shown in Figure 1c, where the measurement electrode locations are highlighted in red. Additionally, following standard measurement protocol, we measured the room and subject skin temperature with a thermal imaging camera (C3, FLIR Systems Inc, Wilsonville, OR, USA). The skin temperature was measured from the midpoint between electrodes 1, 3, 5, and 7, and the room temperature from a table surface close to the measurement setup. Measurements were performed right before and after the impedance measurement. These temperature measurements are plotted in Figure 1d. The room temperature stayed relatively stable during the measurements, and even though some subjects had slight deviations in skin temperature, we did not find a correlation between temperature and the impedance measurements.

### 2.3 Magnetic Resonance Imaging

T1 and T2 weighted magnetic resonance (MR) images of the subjects' right forearm were acquired using a 3 T MRI scanner (Magnetom Skyra; Siemens Ltd, Erlangen, Germany). The following imaging parameters were used for the sequences. T1: TR/TE/FA/voxel size = 981 ms/21 ms/140°/0.625 × 0.625 × 5.5 mm and T2: TR/TE/FA/voxel size = 5130 ms/65 ms/120°/0.547 × 0.547 × 5.5 mm. Since the impedance measurements and MRI were performed in separate sessions, a second 3D surface scan was taken right before the MRI. The purpose of this scan was to locate the electrode positions on the final forearm models (Section 2.4). The subjects were asked to keep the forearm in a similar posture as in the impedance measurements. Three MRI skin markers (LiquiMark 8 mm round MRI markers, Suremark, Mesa, AZ, USA) were placed on the subject's forearm. These skin markers can easily be located on both the T1‐ and T2‐weighted MR images and the surface scans. This data was measured at the AMI Centre, Aalto NeuroImaging, Aalto University School of Science.

### 2.4 Constructing the Forearm Models

The acquired MR images were manually segmented into four tissue types: bone, fat, muscle and blood. The MRI markers were also segmented. The tendons and muscles of the forearm were considered as a single tissue type. Similarly, cortical‐ and cancellous bone were segmented as a single bulk tissue. The skin was not segmented since it was poorly visible in most of the images and obscured by a chemical shift artefact, typical for spin echo sequences. These segmented forearms were then imported into MATLAB (MATLAB 2021) and voxelized using cubic voxels with a side length of 1.1 mm. The skin was added onto the boundaries of the voxelized models as a single voxel layer.

**Figure 2:**
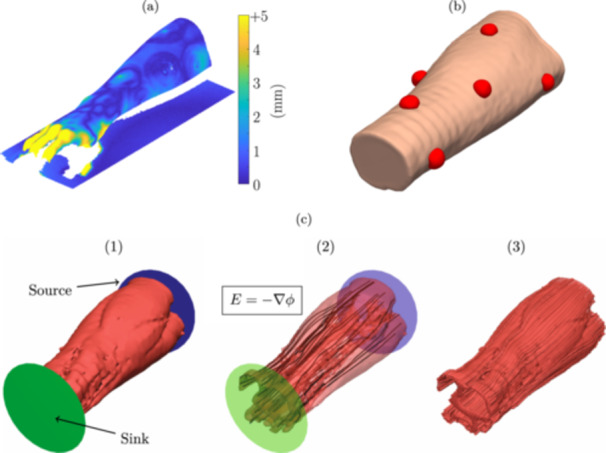
Data from an example subject: (a) distance between the two 3D surface scans and (b) surface representation of voxelized forearm model with the modelled measurement electrodes shown in red. (c) How muscle fibre direction was estimated: (1) a voltage source and sink were placed at the ends of the muscle tissue; (2) the electric field direction was solved inside this volume; and (3) the direction of the electric field was interpreted as the direction of muscle fibres.

The measurement electrode locations, for each individual model, were obtained using both the surface scan taken after the impedance measurements and the surface scan taken before the MRI. First, these scans were fitted together using the dorsal surface of the forearms and the table surface. The table surface reduces this fitting procedure to a simple 2D rotation and translation. Figure 2a shows the distance between the fitted scans for an example test subject. The largest deviations between the scans are found in the hand, which is not included in the models. The electrode adhesive tape is also visible since it has a finite thickness. Second, the segmented MRI markers were fitted to the scanned MRI markers, revealing the measurement electrode locations on the voxelized forearms. The electrodes were modelled as hemispheres, with a radius of 9 mm, resembling the size of the measurement electrode gel. An example forearm, with the electrodes in red, is shown in Figure 2b.

A final modification to the forearm models was made by estimating the direction of muscle fibres. The method used relies on assuming that the Laplace equation produces a flow that resembles the direction of skeletal muscle fibres. This has been shown to provide good estimates in individually segmented muscle models (Choi and Blemker 2013; Handsfield et al. 2017). Here the method was applied to the entire volume of segmented muscle. First, the muscle tissue was modelled as a volume with homogeneous conductivity, with a planar source and sink placed at the ends (Figure 2c (1)). Second, the electric field, resulting from a potential difference between the planes, was solved with our in‐house FEM solver in Section 2.5.1 (Figure 2c (2)). Finally, the direction of the electric field was interpreted as the direction of the muscle fibres (Figure 2c (3)).

### 2.5 Estimating Tissue Conductivities

#### 2.5.1 Forward Problem

Estimating tissue conductivities using impedance data measured from the skin surface requires solving an inverse problem. However, we start by first modelling the impedance, i.e. solving an electrostatic forward problem. Under the quasi‐static approximation (Wang and Eisenberg 1994), valid below the highest frequency considered here (1 MHz), the electric scalar potential (*ϕ*) induced by electric stimulation can be described by the following equation and boundary conditions:

(1) $$\nabla \cdot \vec{\vec{\sigma }}\cdot \nabla \;\phi =0,\;\mathrm{in \Omega},$$

(2) $$\left\{\begin{array}{ll} \vec{n}\cdot \vec{\vec{\sigma }}\cdot \nabla \;\phi =0, & \mathrm{on \delta}\Omega_{N},\;\\ \phi =V_{i}, & \mathrm{on}\;{\delta \Omega }_{i},\;i=1,2. \end{array}\right.$$

Here Ω is the domain (forearm model) and $\vec{\vec{\sigma }}$ the conductivities assigned to each tissue, $\delta \Omega_{N}$ is the boundary of the domain (surface of the forearm model), ${\;\delta \Omega }_{i}$ the current feeding electrodes and $V_{i}$ their potential values. The scalar potential equation can be solved numerically using our in‐house solver based on FEM (Laakso and Hirata 2012). This solver operates on cubical elements and is capable of handling fully anisotropic material parameters (Kataja et al. 2023).

If we know the scalar potential inside the domain, the impedance between two points, for example points $r_{p}$ and $r_{m}$, can be modelled by dividing the potential difference with the current:

(3) $$\bar{Z}\left(\sigma \right)=\frac{\left|\phi \left(r_{p}\right)-\phi \left(r_{m}\right)\right|}{I},$$

where the electric current (I) can be calculated by integrating the normal component of the current density ($J=-\vec{\vec{\sigma }}\cdot \nabla \;\phi$) over a cross‐section plane between the current feeding electrodes. Notice here that the resulting impedance $\bar{Z}$ is a function of the conductivities (σ) used to derive ϕ.

#### 2.5.2 Estimation Method

The optimal conductivities that best describe the measured impedance can be found by minimizing the distance between modelled impedances and measured impedances. However, if we assume that our system contains noise, we can construct a measurement model as follows:

(4) $$Z_{i}={\bar{Z}}_{i}\left(\sigma \right)+n_{i},$$

where $Z_{i}$ is an impedance measurement and ${\bar{Z}}_{i}\left(\sigma \right)$ is the corresponding modelled impedance and $n_{i}∼N\left(0,K\right)$.

**Figure 3:**
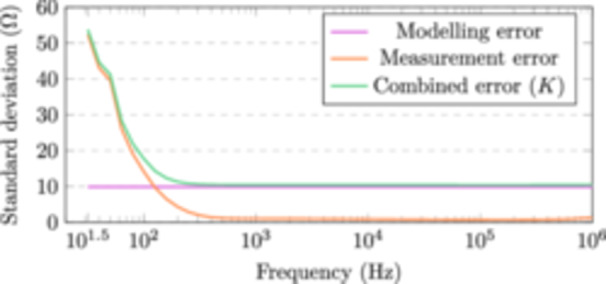
Error from reciprocal impedance measurements, modelling the impedance and their combination.

The noise parameter K can be approximated by combining noise found in the impedance measurements and from modelling the impedance, i.e., solving the forward problem. The impedance measurements contain 35 reciprocal measurements (where the current feeding and voltage sensing electrodes are swapped) for each test subject. In theory, the measured impedance should be the same when measuring the impedance with a reciprocal electrode configuration. The sample standard deviation of the error (measurement error) between these reciprocal measurements is shown in Figure 3. Using simplified models of the forearm to solve the forward problem also introduces an unknown error term into our system (modelling error). Based on our previous study (Kangasmaa and Laakso 2022) we deduced the standard deviation of the modelling error to be 10 Ω over the entire frequency range. Note that these 35 reciprocal measurements were not included in the following analysis, reducing the number of impedance measurements per test subject to 105.

Since we assume that the combined noise from the reciprocal measurements and forward modelling follows the Gaussian distribution, the measured impedance has a probability density function in the form of:

(5) $$P\left(Z_{i}\right)=P\left(Z_{i}|\sigma \right)=\frac{1}{K\sqrt{2\pi }}\text{exp}\left(-\frac{{\left(Z_{i}-{\bar{Z}}_{i}\left(\sigma \right)\right)}^{2}}{2K^{2}}\right).$$

We are interested in finding conductivities given all the measured impedances from all subjects (N = 105 × 10 = 1050). We can express the likelihood of all the impedance measurements, $Z={\left[Z_{1},\ldots ,Z_{N}\right]}^{T}$, given conductivity as:

(6) $$P\left(Z|\sigma \right)=\prod_{i=1}^{N}P\left(Z_{i}|\sigma \right)={\left(\frac{1}{K\sqrt{2\pi }}\right)}^{N}\text{exp}\left(-\frac{1}{2K^{2}}{\left\|Z-\bar{Z}\left(\sigma \right)\right\|}^{2}\right).$$

This is called the likelihood function for the conductivity, describing the probability of the measured impedance given the conductivity. According to Bayes' theorem, we can determine the probability of the conductivity given the measured impedance, i.e. the posterior probability for the conductivity:

(7) $$P\left(\sigma |Z\right)=\frac{P\left(Z|\sigma \right)P\left(\sigma \right)}{P\left(Z\right)},$$

where the term P(σ) is called the prior distribution and the term P(Z) the marginal distribution.

**Figure 4:**
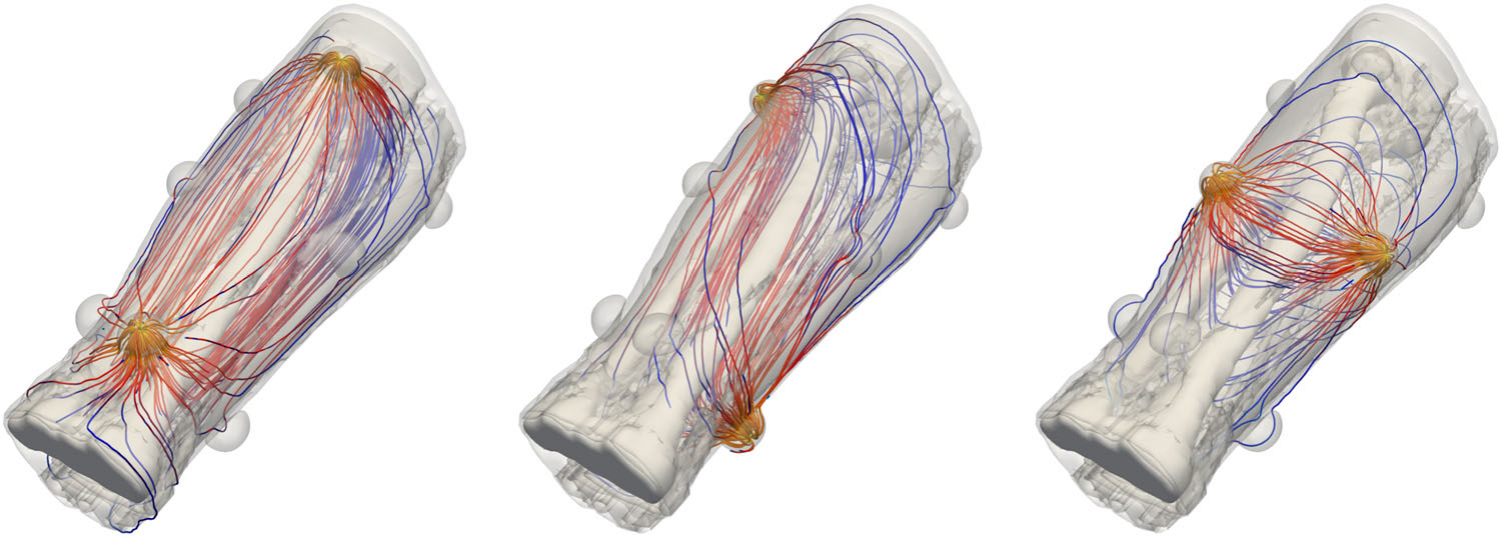
Streamline visualization of the modelled current flow in three different current feeding configurations.

Here we used the following brute force approach to determine the posterior distribution for the conductivities of lateral‐ and longitudinal muscle and fat. All unique impedance measurements were first modelled with all possible combinations for the conductivity according to the values defined in Table 1 to create a lookup table of modelled impedances. Bone, blood, skin, and electrode gel were assigned constant conductivities since our preliminary investigations showed that the method was not sensitive enough to determine their conductivities. For the impedance calculations, conductivities were first assigned to the forearm models (resolution 1.1 mm × 1.1 mm × 1.1 mm), after which the forearm models were coarsened (averaged) to 4.4 mm × 4.4 mm × 4.4 mm. The coarsened models are still anisotropic. This averaging was done to reduce the total computational cost. Figure 4 shows three example calculations where, from left to right, current is fed between electrodes: 1–5, 2–6 and 3–7. The resulting impedances were extremely smooth as a function of conductivity, thus the lookup table was interpolated to a finer resolution of 0.01 S/m. If we assume that the conductivities are found in the lookup table and each combination for the conductivity is equally likely, the prior P(σ) is a constant function over the lookup table.

**Table 1:** Impedance lookup table as a function of conductivity.

Tissue	*σ*_min_	*σ*_max_	Δ*σ*	Δ*σ*_final_
Muscle ⊥	0.02	1.10	0.02	0.01
Muscle ∥	0.30	1.00	0.02	0.01
Fat	0.02	0.60	0.02	0.01
Bone	constant 0.01			
Blood	constant 0.70			
Skin	constant 0.44			
Electrode gel	constant 1.60			

*Note:* The lookup table does not depend on frequency.

Since we now know the likelihood (6) and prior probability at each discrete point in the lookup table, the marginal distribution reduces to a simple summation over all possible conductivity values:

(8) $$P\left(Z\right)=\sum_{\sigma }P\left(Z|\sigma \right)P\left(\sigma \right).$$

With the likelihood, prior and marginal distribution calculated, we attain a 3D posterior distribution for the conductivity of lateral‐ and longitudinal muscle and fat given all measured impedances from all test subjects. As the reader might note, we have yet to mix frequency into any equation. The modelled impedances do not depend on frequency; thus, the constructed lookup table can be used to calculate the posterior distribution (7) at any of our measurement frequencies ($f=\left[10^{1.5},10^{1.6}\ldots ,10^{6}\right]$ Hz).

#### 2.5.3 Conductivity Statistics and Model Evaluation

In our Bayesian approach, conductivity values are treated as random variables and therefore are described by probability distributions. The main statistics that we calculate from the estimated posterior distribution are the maximum a posteriori (MAP) estimate and 95% credible intervals for the marginal distributions of the conductivities. The MAP estimate corresponds to the mode of the posterior distribution. The 95% credible intervals describe limits which contain the true parameter value with a 95% probability. Here, these limits are equal‐tailed.

The robustness of the conductivity estimates was evaluated using leave‐one‐out cross‐validation (LOOCV). The posterior distribution and corresponding MAP estimates were calculated by leaving one subject out of each round of calculation. The coefficient of determination ($R^{2}$) was calculated for the subject that was left out with the new MAP estimates:

(9) $$R^{2}=1-\frac{\sum_{i=1}^{N}{\left(Z_{i}-{\bar{Z}}_{i}\left(\sigma_{\text{MAP}-\text{LOOCV}}\right)\right)}^{2}}{\sum_{i=1}^{N}{\left(Z_{i}-\frac{1}{N}\sum_{j=1}^{N}Z_{j}\right)}^{2}},$$

where the summations are over all measurements of the subject that was left out and $\sigma_{\text{MAP}-\text{LOOCV}}$ is the conductivity MAP estimate calculated using the data of all other subjects.

### 2.6 Propagation of Uncertainty From Conductivity to Induced Electric Fields

**Figure 5:**
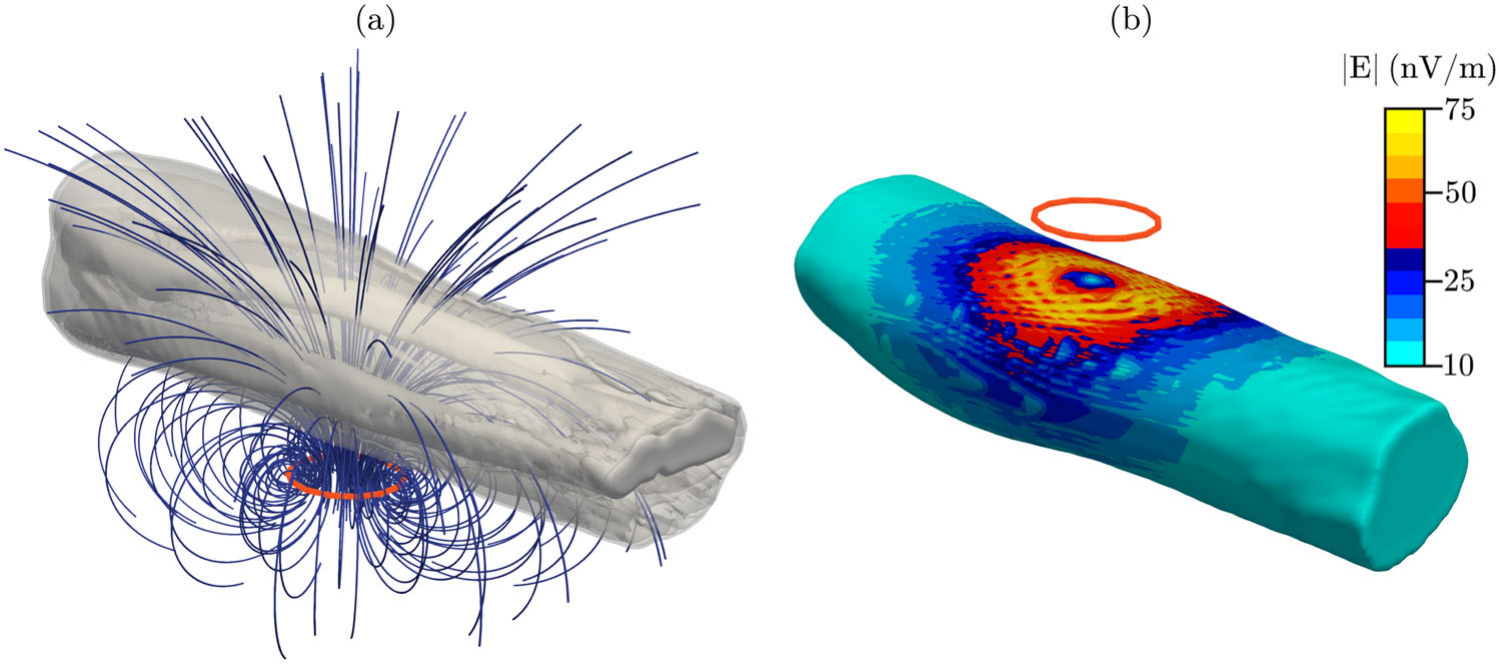
(a) Magnetic field produced by the coil. (b) Averaged induced electric field 1.1 mm under the skin surface (MAP values at 1 kHz).

We used numerical dosimetry modelling and random sampling to study how the uncertainty in the newly estimated conductivity values propagates into induced electric fields. First, we created a localized magnetic field exposure scenario. In this scenario, a circular single‐loop coil, with a radius of 2 cm, was placed 2 cm under the midpoint of one of the forearm models tangential to its surface. A 1 A/s current was modelled through the coil in all following calculations. The same in‐house FEM solver as in 2.5.1 was used to calculate the induced electric field $E=-\nabla \phi -\frac{\partial A}{\partial t}$. The source term in Equation 2 was changed from a voltage source to the magnetic vector potential induced by the coil, which was calculated with the Biot‐Savart law. The forearm model used for the exposure scenario, circular coil, and the resulting magnetic field is illustrated in Figure 5a.

Second, 1000 conductivity samples were randomly drawn from the 3D posterior distributions at six frequencies: 50 Hz, 100 Hz, 1 kHz, 10 kHz, 100 kHz and 1 MHz. These sampled conductivity values were assigned to the forearm model. The conductivity of bone, blood and skin were assigned the same constant values as in Table 1. Here, we used the models’ original resolution (1.1 mm × 1.1 mm × 1.1 mm) to perform the calculations. The induced electric field was then calculated and averaged over a 2.2 mm × 2.2 mm × 2.2 mm cube in each tissue. Since the skin was modelled as a 1.1 mm thick layer, the averaging cube was allowed to overlap with underlying tissues as recommended by ICNIRP (2010). Both the maximum value of the induced electric field and the 99.9th percentile value were calculated in each distinct tissue compartment. This results in sampling distributions for both the maximum and 99.9th percentile values at six frequencies. Figure 5b shows the averaged induced electric field, calculated with the MAP values at 1 kHz. The field has been interpolated to a surface 1.1 mm below the skin surface.

## 3 Results

### 3.1 Forearm Models

**Figure 6:**
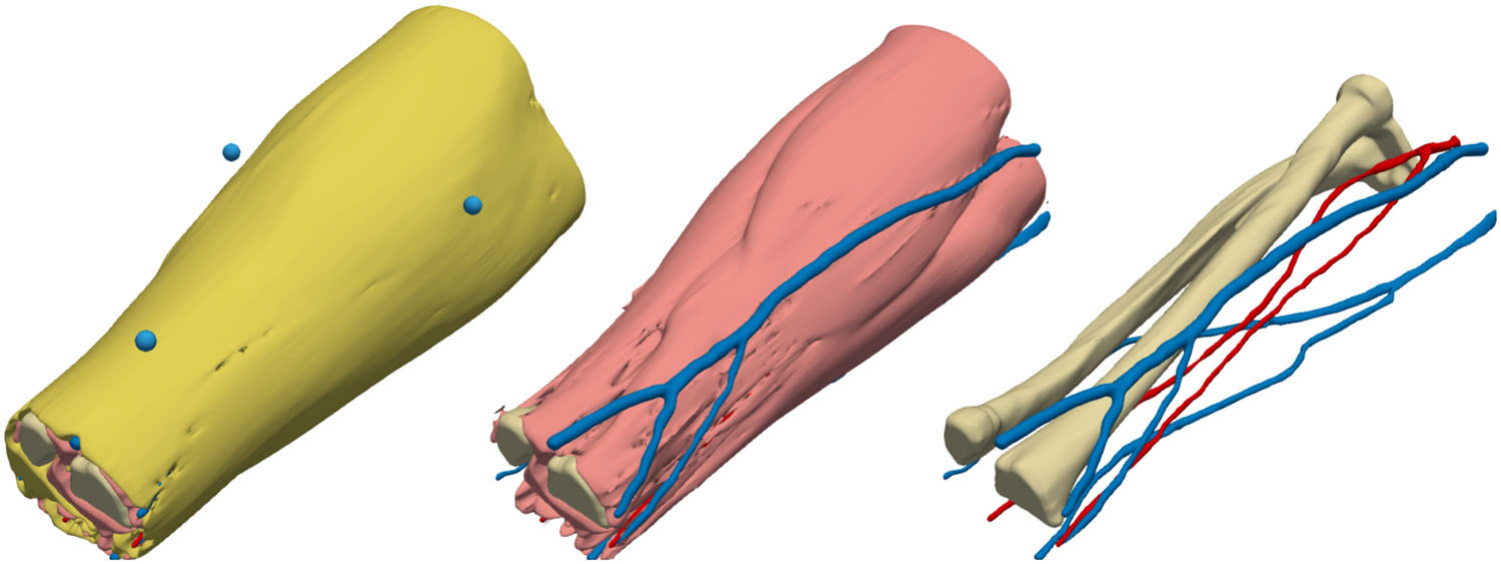
Surface models of fat (yellow), MRI markers (cyan), muscle (pink), bone (beige), veins (blue) and arteries (red).

The subject‐specific forearm models represent the human forearm as simplified voxelized volumes of five different tissue types: blood, bone, fat, anisotropic skeletal muscle and skin. The largest forearm model contains 1,065,713 and the smallest 555,275 (mean 759,234) non‐air cubical voxels, with a side length of 1.1 mm. Figure 6 shows surface representations of the model tissues from an example test subject. The mean volume proportion and sample standard deviation of each tissue over all 10 forearm models are listed in Table 2. Naturally, the largest variations are found in the amount of fat and muscle tissue.

**Table 2:** The mean volume proportion and standard deviation of tissues in the forearm models.

Tissue	Mean (%)	Std (±%)
Blood	1.3	0.2
Bone	10.6	1.4
Fat	22.0	6.6
Muscle	60.4	5.5
Skin	5.8	0.5

*Note:* The mean volume proportion was calculated by dividing each tissue's volume by the total volume of the forearm, after which the mean and standard deviation were calculated over all 10 models.

### 3.2 Estimated Tissue Conductivities

**Figure 7:**
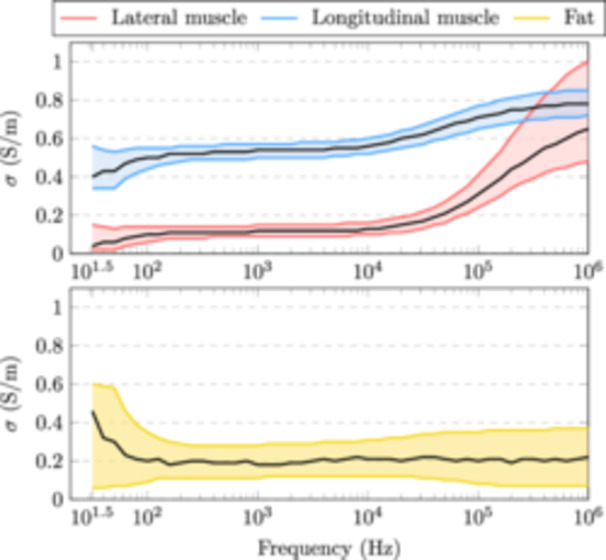
Estimated conductivities for lateral muscle, longitudinal muscle and fat. The MAP estimate is shown in black and the 95% credible intervals in colour. These values are also provided in table format as supplementary material (Conductivity_table.xlsx).

The estimated conductivities for lateral muscle, longitudinal muscle and fat are shown in Figure 7. For each tissue, the MAP estimate is plotted in black and the 95% credible intervals in colour. These results have been derived from posterior distributions for the conductivity, independently at all 46 logarithmically spaced measurement frequencies, from 31.6 Hz to 1 MHz.

**Figure 8:**
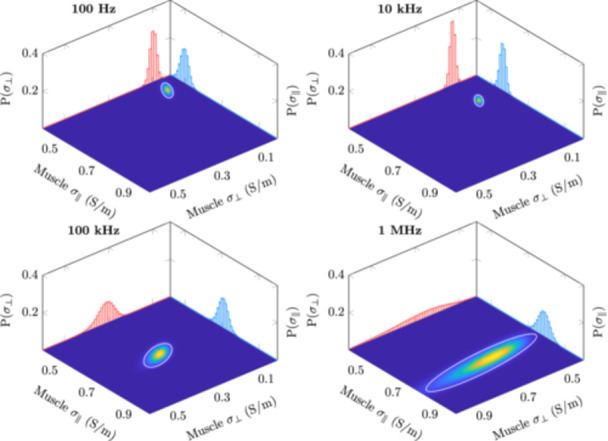
Normalized 2D marginal distributions for lateral (*σ*_*⊥*_) and longitudinal muscle (*σ*_*∥*_) visualized as heatmaps. The colour intensity at a particular point indicates the probability of that conductivity combination occurring simultaneously. The white ellipse shows the area where the conductivity is found with 90% probability. The smaller the area, the greater the certainty of the estimates. The projected plots show the 1D marginal distribution for *σ*_*⊥*_ (red) and *σ*_*∥*_ (blue).

**Figure 9:**
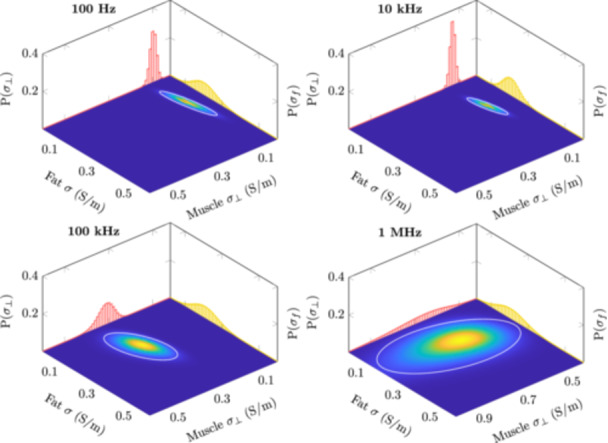
Normalized 2D marginal distributions for lateral muscle (*σ*_*⊥*_) and fat (*σ*_*f*_) visualized as heatmaps. The colour intensity at a particular point indicates the probability of that conductivity combination occurring simultaneously. The white ellipse shows the area where the conductivity is found with 90% probability. The smaller the area, the greater the certainty of the estimates. The projected plots show the 1D marginal distribution for *σ*_*⊥*_ (red) and *σ*_*f*_ (yellow).

**Figure 10:**
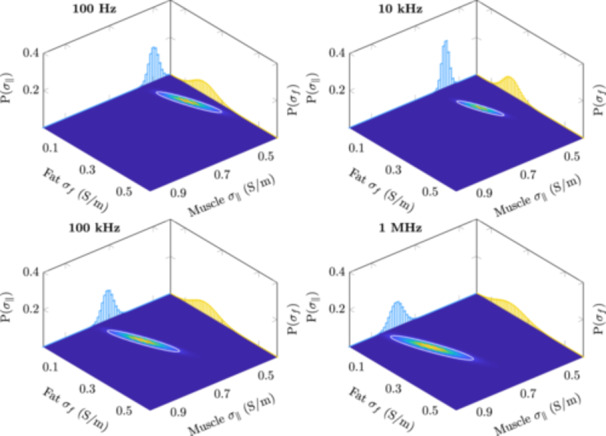
Normalized 2D marginal distributions for longitudinal muscle (*σ*_*∥*_) and fat (*σ*_*f*_) visualized as heatmaps. The colour intensity at a particular point indicates the probability of that conductivity combination occurring simultaneously. The white ellipse shows the area where the conductivity is found with 90% probability. The smaller the area, the greater the certainty of the estimates. The projected plots show the 1D marginal distribution for *σ*_*∥*_ (blue) and *σ*_*f*_ (yellow).

The 3D posterior distribution is difficult to visualize. However, we can observe marginal distributions in 2D as heatmaps and 1D as plots. These distributions are shown in Figures 8, 9, 10 for lateral‐ and longitudinal muscle, lateral muscle and fat, and longitudinal muscle and fat, respectively. In these heatmap representations, the 2D marginal probability has been normalized, thus a smaller area corresponds to a more precise estimate. Additionally, an ellipse, plotted in white, has been fitted to the 2D marginal probability so that it encases 90% of the probability mass. The 1D plots have not been normalized, showing the probability of discrete conductivities. Notice that, in Figures 8 and 9, the scaling of the conductivity axis changes in the 1 MHz plots.

### 3.3 Cross Validation of Estimated Conductivities

**Figure 11:**
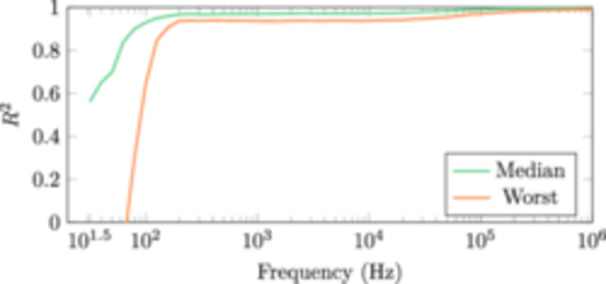
The median and worst $R^{2}$ calculated with the LOOCV MAP estimates.

**Figure 12:**
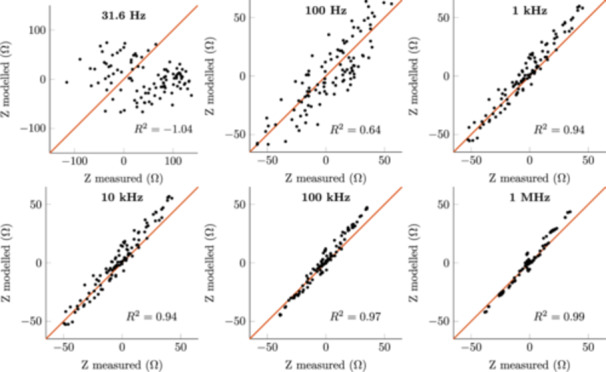
Modelled impedances versus the measured impedances from the worst subject calculated with the LOOCV MAP estimates.

The robustness of the conductivity estimates was evaluated using LOOCV. Figure 11 shows the median and worst $R^{2}\;$calculated with the LOOCV MAP estimates. The worst $R^{2}$ degrades sharply at roughly 100 Hz, even becoming negative below 63 Hz. This is further illustrated in Figure 12 where all the measured impedances are plotted against the corresponding modelled impedances from the worst subject, at six different frequencies. The line of the best possible fit, i.e. the measured and modelled values are the same, is plotted in red for reference. At the lowest measurement frequency, the modelled impedances fit the measured data very poorly. As the frequency increases, the fit gets increasingly better.

### 3.4 Effect of Uncertainty in Conductivity on Induced Electric Fields

**Figure 13:**
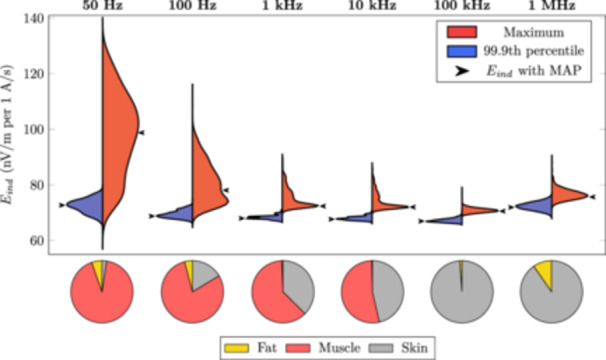
Normalized kernel density plots of the averaged induced electric field strength (*E*_*ind*_) calculated with 1000 random conductivity samples at six frequencies. The arrows indicate the induced electric field that would result from using the MAP estimate. The pie charts show in which tissue the maximum induced electric field was found.

Figure 13 illustrates how the uncertainty in the estimated conductivity values propagates into induced electric field strengths when a forearm model is exposed to a localized magnetic field. The distributions for both the 99.9th percentile and the maximum value of the averaged induced electric field have been plotted using kernel density estimation with a Gaussian kernel. All distributions have been normalized. Arrows are plotted to indicate the electric field strength that would be induced if the MAP estimate of the conductivities was used. The pie charts show in which tissue the maximum electric field value appears. The 99.9th percentile value appeared in the skin in all 1000 samples at every considered frequency.

### 3.5 Parametric Model of Conductivities

A common way to present tissue dielectric data is with relaxation models. A popular model used to describe the properties of human tissues is the Cole–Cole model (Cole and Cole 1942). The Cole–Cole model was originally formulated for the complex dielectric constant, but can also be written for the complex‐valued conductivity:

(10) $$\sigma^{*}=\sigma_{\infty }+\frac{\sigma_{\text{DC}}-\sigma_{\infty }}{1+{\left(j\omega \tau \right)}^{1-\alpha }},$$

where $\sigma_{\text{DC}}$ and $\sigma_{\infty }$ are the low‐frequency and the high‐frequency electrical conductivity values, τ is the relaxation time constant, and α (0 < α < 1) the Cole–Cole exponent.

**Figure 14:**
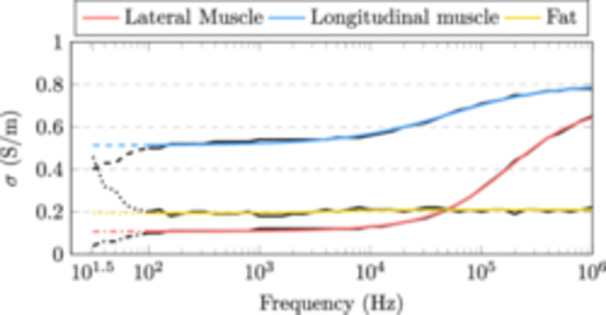
The real part of the Cole–Cole fitted conductivity plotted the MAP estimates (in black).

The model was fitted to the MAP estimate of each tissue by using a nonlinear least squares optimization routine. Only the real part of the complex impedance was used for the fitting. The real part of the Cole–Cole fitted complex conductivities are plotted in Figure 14. For comparison, the MAP estimates for each tissue are plotted in black. Values below 100 Hz were not used for the fitting since both the reciprocal measurement error and $R^{2}$ values imply that the data is unreliable at those frequencies. The corresponding Cole–Cole model parameters are also presented in Table 3. Notice that τ and α parameters for fat have a very small effect on the fit and are thus unreliable.

**Table 3:** Cole–Cole parameters for each tissue.

Tissue	*σ*_DC_	*σ*_∞_	τ(μs)	α
Lateral muscle	0.107	0.711	0.901	0.198
Longitudinal muscle	0.513	0.808	3.099	0.318
Fat	0.194	0.209	38.367	0.000

*Note:* These values are valid between 100 Hz–1 MHz.

## 4 Discussion

In this work, we improved upon a method (Kangasmaa and Laakso 2022) to derive human in vivo tissue conductivities using noninvasive impedance measurements and computational modelling. The conductivity was estimated for anisotropic skeletal muscle and fat, from 100 Hz to 1 MHz. Additionally, a probabilistic approach was used to derive statistics for these conductivity estimates.

First, it should be stated that the derived electrical conductivity values presented in this work are not the absolute conductivities of these tissues, but rather conductivities that should be used to model the interactions between the human body and electromagnetic fields at low frequencies. The MAP estimates are also an arbitrary choice as the best value. For example, in Figure 9, we can see that at 1 MHz the marginal distributions for lateral muscle and fat are very wide, meaning that the MAP estimate has large uncertainty. In turn, if the marginal distributions are narrow, like with muscle at low frequencies, the discrete values at which the conductivities have been estimated (resolution of 0.01 S/m) become the limiting factor. Moreover, the conductivity estimates below 100 Hz are to be discarded. Both the measurement error, from reciprocal measurements, and $R^{2}$, from our LOOCV calculations, show large uncertainties at these extremely low frequencies. This is likely due to a low signal‐to‐noise ratio of our impedance measurements at these frequencies. Despite these shortcomings, the presented 95% credible intervals provide clear conservative limits from which to choose appropriate conductivities.

**Figure 15:**
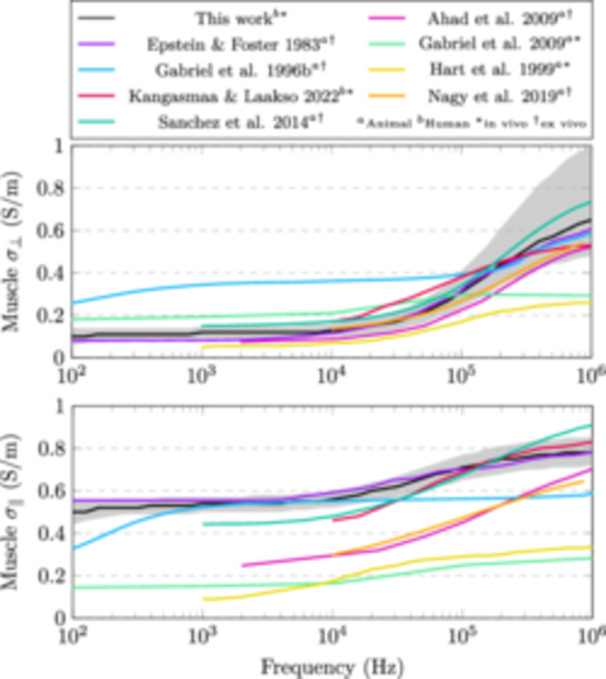
Comparison of muscle conductivities in the lateral (*σ*_*⊥*_) and longitudinal (*σ*_*∥*_) directions. The grey shaded area shows the 95% credible intervals of our estimates.

The conductivity of muscle was found to be highly anisotropic at frequencies below 10 kHz, with the ratio of lateral to longitudinal conductivity being approximately 1:5. This anisotropy was also shown to decrease with increasing frequency. In Figure 15, values from literature (A Ahad et al. 2009; Epstein and Foster 1983; Gabriel, Lau, and Gabriel Peyman, and Grant 2009, 1996; Hart, Berner, and McMillen 1999; Kangasmaa and Laakso 2022; Nagy et al. 2019; Sanchez et al. 2014) are compared to those found in this study. Our results coincide well with those of Epstein and Foster (1983) who measured the dielectric properties from excised dog skeletal muscle at body temperature. The rather large discrepancies between studies may be attributed to differences in measurement method, sample species and preparation, and temperature. Nevertheless, almost all of these studies show that the conductivity of muscle increases and the anisotropy decreases with increasing frequency. This is likely due to the manifestation of β‐dispersion, which is related to charges accumulating at cell membrane surfaces (Maxwell‐Wagner effect) (Kuang and Nelson 1998). However, making conclusions about microscopic structures using macroscopic models is to be taken with a grain of salt.

It is worth noticing that our new conductivity estimates for skeletal muscle differ slightly from our previous study (Kangasmaa and Laakso 2022). In that work, we were limited to a diagonal‐matrix approximation for the anisotropic conductivity tensor used to represent skeletal muscle conductivity. The fibre direction of muscle tissue was also assumed in the direction of the principal axis of our models. In the present work, we paid special attention when modelling muscle fibre direction. Using muscle geometry and Laplacian vector field simulations has been shown to provide an alternative approach to computationally model skeletal muscle fibre directions (Choi and Blemker 2013; Inouye, Handsfield, and Blemker 2015; Saito, Zhou, and Kavan 2015; Handsfield et al. 2017). It is to be noted that all aforementioned works performed their simulations on models of individual muscles, compared to this work where we used the entire muscle mass of the forearm. Even with this simplification, we can qualitatively say that this results in a better estimate than assuming the fibre direction parallel to the principal axis of the forearm.

The conductivity of fat was found to be roughly a constant 0.2 S/m over the frequency range considered. However, the 95% credible intervals are wide making the estimate quite uncertain. Nevertheless, we can compare our new estimates to previous studies, where the conductivity of fat at 10 kHz has been shown to be: 0.02 S/m (Gabriel, Lau, and Gabriel 1996); 0.08 S/m Kangasmaa and Laakso (2022); 0.09 S/m (Gabriel, Peyman, and Grant 2009); 0.15 S/m (Wake, Sasaki, and Watanabe 2016); 0.21 S/m (Smith and Foster 1985); and 0.41 S/m (Stoneman et al. 2007). In all these works, fat shows little to no β‐dispersion below 1 MHz. Smith and Foster (1985) found large variations in bone marrow and adipose tissue conductivities as a function of water content, which may be the source of variations between studies. The authors also note that due to the heterogeneity of these tissues, they should be described with a range rather than a single value. Our wide credible intervals seem to capture this variation. Additionally, our new estimates should correspond to high water‐content fat tissues since the tissue is alive in its normal functioning state. Our values are also similar to those found by Hershkovich et al. (2019) (0.20 S/m at 100 kHz) who measured the conductivity of fat using skin folds from live human subjects. Unfortunately, they fail to report the uncertainty for their estimate.

A major benefit of using a probabilistic approach to derive the conductivity estimates is that the results are in the form of probability distributions. Random sampling from a probability distribution offers an easy and intuitive way to incorporate uncertainties in model input parameters to quantify uncertainty in model outputs. Here we explored how uncertainty in the estimated tissue conductivities propagates to induced electric fields when a forearm is exposed to a magnetic field from a single loop coil. Below 1 kHz, the maximum value of the electric field has a wide probability distribution indicating large uncertainty due to the conductivity values. The uncertainty reduces at higher frequencies, where the conductivity values are less uncertain. The 99.9th percentile value of the induced electric field has considerably lower uncertainty and may even be a reliable measure below 100 Hz. The results can also be used to estimate the uncertainty in the induced electric field values when using the MAP estimate of the conductivity (Figure 13).

The Cole–Cole model fits the MAP estimates extremely well. Thus, when extracting conductivity values from this study it is recommended to use the Cole‐Cole model (10) and the derived constants supplied in Table 3. The uncertainty for these estimates can be obtained from the posterior probability distributions (Figures 8, 9, 10). Only the real part of the complex‐valued conductivity was used when fitting the MAP estimates to the model. Thus, drawing conclusions about the complex part of the complex conductivity, i.e. permittivity, can be only done at one's own discretion. This caution also applies to extrapolating values below 100 Hz or above 1 MHz.

This study has some limitations. Firstly, the anatomical forearm models represent a simplified structure of the true forearm anatomy. Secondly, bone, blood and skin were assigned frequency‐independent constant conductivities since our method was not sensitive enough to determine their conductivities. This was likely due to the low volume of these tissues. Third, at extremely low frequencies the impedance measurements suffered from a low signal‐to‐noise ratio, which limited our reliable frequency range to 100 Hz–1 MHz. Finally, it is worth noting that the data collection and model creation is a rather complex and time‐consuming procedure. However, the method could be applied to more simple models, where the results of this study may be used as guiding limits, increasing the applicability of these results and the proposed method.

## 5 Conclusion

In this study, noninvasive impedance measurements were combined with subject‐specific forearm models to computationally estimate the conductivity of anisotropic skeletal muscle and fat. The most likely value for the conductivity of fat was found to be on average 0.20 S/m from 100 Hz to 1 MHz. In the same range, skeletal muscle conductivity increased from 0.10 to 0.65 S/m in the lateral direction and 0.50 to 0.78 S/m in the longitudinal direction. These estimates were accompanied with robust uncertainty bounds. These results provide the latest information on the electrical properties of live human tissue, most notably at frequencies below 10 kHz, where previous knowledge is lacking and most uncertain.

## Ethics Statement

All subjects gave their written informed consent, and the study was approved by the Aalto University Research Ethics Committee (diary number D/1545/03.04/2023).

## Conflicts of Interest

The authors declare no conflicts of interest.

## Supporting information

Supporting information.
